# Selenium- and/or copper-substituted hydroxyapatite: A bioceramic substrate for biomedical applications

**DOI:** 10.1177/08853282231198726

**Published:** 2023-08-21

**Authors:** Sara I Korowash, Zalike Keskin-Erdogan, Bahaa A Hemdan, Lady V Barrios Silva, Doreya M Ibrahim, David YS Chau

**Affiliations:** 1 Department of Refractories, Ceramics and Building Materials, 68787 National Research Centre, Cairo, Egypt; 2 Division of Biomaterials and Tissue Engineering, Eastman Dental Institute, Royal Free Hospital, 4919 UCL, London, UK; 3 Chemical Engineering Department, Imperial College London, London, UK; 4 Department of Water Pollution Research, 68787 National Research Centre, Cairo, Egypt

**Keywords:** Selenium, copper, osteoblasts, sulphur, tissue engineering, antimicrobial

## Abstract

Atomic substitution or doping of a bioceramic material hydroxyapatite (HA) with specific ions is an appealing approach for improving its biocompatibility and activity, as well as imparting antibacterial properties. In this study, selenium- and/or copper-substituted hydroxyapatite powders were synthesized by an aqueous precipitation method and using the freeze-drying technique. The molar concentrations of constituents were calculated based on the proposed mechanism whereby selenium (Se^4+^) ions partially substitute phosphorus (P^5+^) sites, and copper (Cu^2+^) ions partially substitute (Ca^2+^) sites in the HA lattice. Dried precipitated samples were characterized using Inductively coupled plasma optical emission spectroscopy (ICP-OES), X-ray diffraction analysis (XRD), Fourier-transform infrared spectroscopy (FTIR) and Field-emission scanning electron microscopy with energy dispersive X-ray spectroscopy (FESEM-EDX). Accordingly, substitution of Se^4+^ and/or Cu^2+^ ions took place in the crystal lattice of HA without the formation of any impurities. The presence of sulphur (S^2-^) ions in the hydroxyapatite was detected by ICP-OES in all samples with copper substituted in the lattice. The cytotoxicity of the powders on osteoblastic (MC3T3-E1) cells was evaluated in vitro. Selenium substituted hydroxyapatite (SeHA), at the concentration (200 μg/mL), demonstrated higher populations of the live cells than that of control (cells without powders), suggesting that selenium may stimulate the proliferation of these cells. In addition, the copper substituted hydroxyapatite (CuHA) and the selenium and copper substituted hydroxyapatite (SeCuHA) at the concentrations (200 and 300 μg/mL) and (200 μg/mL), respectively demonstrated better results than the unsubstituted HA. Antimicrobial activity was assessed using a well-diffusion method against *Streptococcus mutans* and *Candida albicans*, and superior results has obtained with SeCuHA *samples*. Presented findings imply that selenium and/or copper substituted modified hydroxyapatite nanoparticles, may be an attractive antimicrobial and cytocompatible substrate to be considered for use in a range of translational applications.

## Introduction

Hydroxyapatite [HA, Ca_10_(PO_4_)_6_(OH)_2_], is the main inorganic component of hard tissues, in biomedical applications HA has a variety of uses in maxillofacial and orthopaedic surgery, dentistry, drug delivery, coatings on metallic prostheses, bone cement, and fillers, because of its excellent biocompatibility, osteoconductivity, bioactivity, nontoxicity and thermodynamic stability.^1–4^

However, pure HA has some limitations, such as poor mechanical strength and lack of antibacterial properties. As such, researchers have focused on modifying HA by incorporating different ions into its crystal lattice to enhance its biological and physicochemical properties. One of the promising approaches to modify HA is atomic substitution or doping with specific ions, which has been shown to improve its biocompatibility, bioactivity, and antimicrobial properties.^5–7^ Selenium (Se) and copper (Cu) are two trace elements that have been of interest in biomedical research due to their therapeutic potential. Selenium has been reported to possess antioxidant, anti-inflammatory, and anticancer properties,^8–11^ while copper is essential for bone formation and has been shown to possess antibacterial properties.^12^

Selenium has been shown to help with cardiovascular disease, cancer, thyroid, brain, bone, tissue reproduction, viral infections, and the immune system.^13^ A series of different concentrations of selenium (between 1 to 5 mol%) substituted hydroxyapatite powders were prepared previously by Korowash et al. (2017) using an aqueous precipitation method. In their study, the cytotoxicity of the powders on both human bone marrow mesenchymal stem cells (BM-MSCs) and umbilical cord-derived mesenchymal stem cells (UC-MSCs) was studied in vitro and in their work in comparison to pure HA powders, 0.592 mM Se, corresponding to a 2 mol% Se showed no cytotoxicity, but stimulated proliferation of UC-MSCs.^14^

The poor antibacterial activity of pure HA limits its long-term stability and increases the risk of implant-related infections and the likelihood of implantation failures.^15^ Therefore, researchers have been exploring different approaches to enhance the antibacterial properties of HA to overcome this challenge. Researches including successfully doped HA with elements as silver (Ag), zinc (Zn), cerium (Ce), manganese (Mn), samarium (Sm), and Cu into HA, prevented implant-related infections.^16,17^ Gomes et al. (2018) has reported that Cu integrated HA presents superior antibacterial activity and least cytotoxicity of all the substitutions.^18^ Moreover, another approach is to incorporate sulphur (S^2-^) or sulphate (SO_4_^2-^) ions into HA. Sulphur and sulphates are known as biological cements because they prevent osteoarthritis and help regenerate skin, hair, nails, and cartilage. They have long been known to have antibacterial properties and are also clinically utilized to treat hypercalcemia.^19–21^ The replacement of sulphate ions in hydroxyapatite had received little attention in research articles.^22^

Previous studies have mostly reported the successful substitution of various ions into HA lattice, including strontium (Sr), magnesium (Mg), Zn and Cu, as a mono or di-substitution, which have been shown to improve its mechanical strength, osteoinductivity, and antibacterial properties.^23–27^ Few studies have also looked into the co-incorporation of Se and Sr or Zn into the HA in an effort to modify its biological characteristics.^28,29^ However, to the best of our knowledge, the co-doping of selenium and copper into HA has never been examined.

From chemical precursors, mainly calcium and phosphorus, hydroxyapatite can be produced using a range of techniques, including dry, wet, thermal, or a mixture of these. As well as HA can be extracted from naturally occurring sources such animal scales and bones, which have large concentrations of HA. Different synthesis techniques result in different crystallinities, sizes, and morphologies.^30–32^

In this study, selenium- and/or copper-substituted hydroxyapatite powders were synthesized by an aqueous precipitation method, while being processed under various conditions, including pH, temperature, time and chemical molar ratios to obtain a pure phase of HA. Following that, a freeze-drying method was used to produce finer material.^33^

The objective of the present work; is to enhance the biocompatibility and antimicrobial properties of synthetic HA by simultaneous ion doping to produce an antimicrobial bioactive material. This material could have a wide range of applications, including toothpaste formulation and use as a filler or in tissue engineering scaffolds to prevent post-implant infections and promote bone/tooth healing.

## Materials and methods

### Preparation of hydroxyapatite powders

Stoichiometric HA nanopowders were prepared through an aqueous precipitation method.^33,34^ As raw materials, calcium carbonate (CaCO_3_, ≥99%) and di-ammonium hydrogen phosphate ((NH_4_)_2_HPO_4_, ≥99%), purchased from Merck KGaA, Germany, were used as a source of Ca^2+^ and PO_4_^3-^ ions respectively, with addition of nitric acid. The molar ratio of Ca/P for the reaction was 1.67 and pH was kept at 8.3 by adding ammonia solution.^33^ Sodium selenite (Na_2_SeO_3_, 99%) and/or copper sulfate pentahydrate (CuSO_4_.5H_2_O, ≥98%), purchased from Merck KGaA, Germany, were added during synthesis to form three different hydroxyapatite phases: selenium substituted hydroxyapatite (SeHA) with selenium percentage (1.96 mol% Se/(P+Se)) at the expense of phosphate salt; copper substituted hydroxyapatite (CuHA) with copper percentage (5 mol% Cu/(Ca+Cu)) at the expense of calcium salt; selenium and copper substituted hydroxyapatite (SeCuHA) with selenium percentage (1.96 mol% Se/(P+Se)) and copper percentage (5 mol% Cu/(Ca+Cu)) at the expense of phosphate salt and calcium salt, respectively, as shown in Table 1. The reaction took place at room temperature then left for 24 h under continuous stirring at 40°C. The formed precipitates were carefully washed with distilled water, frozen at –20°C and dried in a lyophilizer.

**Table 1.** The constituents molar concentration and ratios in the different prepared samples.

Samples	Molar concentration of constituents				(Ca+Cu)/(P+Se) molar ratio	Cu/(Ca+Cu) (%)	Se/(P+Se) (%)
	Ca	P	Se	Cu		
HA	5	3	—	—	1.67	—	—
SeHA	5	2.9412	0.0588	—	1.67	—	1.96
CuHA	4.75	3	—	0.25	1.67	5	—
SeCuHA	4.75	2.9412	0.0588	0.25	1.67	5	1.96

### Powders characterization

The elemental composition of the prepared powder samples was determined by ICP-OES (ICP-OES, Agilent 5100 Synchronous Vertical Dual, Australia). The molar ratio of calcium to phosphorus and the concentration of added Se and Cu, in addition to S that was assumed to be incorporated into HA by using the copper source CuSO_4_.5H_2_O, were calculated from the results obtained.

In addition, phase identification of the HA samples was evaluated using X-ray diffractometer (XRD) (Bruker D8 diffractometer, Germany) utilizing a 40 kV accelerating voltage, 35 mA, and Cu Kα radiation (λ = 1.54184 Å).

Fourier-transform infrared (FTIR) spectroscopy (Spectrum One, Perkin Elmer, UK) was used to identify the functional groups of HA samples. The spectral area analysed was over the range of 4000– 500 cm^−1^ with a resolution of 4 cm^−1^, and the initial background absorbance was collected for calibration.

The surface morphology of powders was investigated under a field emission scanning electron microscope (FESEM) using a Zeiss Sigma 300 VP FESEM equipped with Zeiss SmartEDX (Zeiss, Cambridge, UK). The powders were dispersed on a clean glass surface, and a stub with sticky 12 mm carbon tabs was gently tabbed from above to collect sample from the glass, and excess was removed using compressed air. Stubs were sputter coated with 95% gold and 5% palladium before inspection.

### Antimicrobial activity study

Antimicrobial efficacy of the HA powder samples was evaluated on representative microbial strains, including *Streptococcus mutans* and *Candida albicans* (ATCC, USA). This was carried out using agar well diffusion assay with Mueller-Hinton agar (MHA) and Sabouraud Dextrose Agar (SDA) media (Sigma-Aldrich, UK).^35^ 100 µL of microbial suspension were uniformly disseminated onto the surface of MHA and SDA, with a sterile cotton swab. Wells of 6 mm in diameter were holed with a cork borer in an inoculated agar then filled with 50 µL of the sample in distilled water suspension. The plates were then left for about 1 day at 37°C for *Streptococcus mutans* and 2 days at 28°C for *Candida albicans* growth. Ciprofloxacin antibiotic was used as a positive control and sterile distilled water was used as a negative control. The experiments were run in three replicates and following incubation, the antimicrobial activity of the samples was assessed by measuring the diameter of the zone of inhibition that formed around the wells.

### In vitro biocompatibility study

The osteoblastic cell line of MC3T3-E1 from mouse was acquired from ECACC in the United Kingdom and cultured in MEM-α medium that supplemented with 10 v/v% FBS and 1 v/v% P/S (Penicillin/Streptomycin). Sample powders (HA, SeHA, CuHA and SeCuHA) suspended in complete growth media in various concentrations (200 and 300 μg/mL) were placed in 96-well tissue culture plates (TCP) after sterilization under UV for 30 min. The cells were seeded on the HA samples or on TCP, then left in an incubator for 3 and 7 days at 37°C and 5% CO_2_. For each sample type and TCP, three replication trials were conducted.

Following the incubation time periods, the metabolic activity of the cells was assessed using the Cell-Titer One reagent/MTS test kit (Promega, Southampton, UK). 20 µL of was added to each well containing 100 µL culture media and incubated at 37°C for 1.5 h. Then, using 120 µL of the media from each well on the new 96-well which was read with a Plate Reader (Tecan Infinite M200, Switzerland), absorbance was determined at 490 nm. As a reagent blank, wells holding only media/media and samples were prepared for each time point.

All values are presented as means and standard deviations (SD) after one-way ANOVA, Holm Comparison Test, Bonferroni's Multiple Post-Test (*p*<0.05) were used to assess the data.

Live-Dead Assay: The LIVE/DEAD^TM^ Kit (Gibco L3224, Thermo Fisher, Paisley, UK) was used to evaluate the in vitro qualitative assessment of cell viability. The cells and cells with samples were exposed to calcein-AM and ethidium homodimer-1 for 30 min at room temperature in the dark after being incubated for 7 days at a density of 1.5 × 10^4^ cells/mL. An inverted fluorescence microscope (LEICA Instruments, Milton Keynes, UK) and image Capture Pro software were used for the examination.

## Results and discussion

The results of the ICP-OES of the apatite powders are shown in Table 2 demonstrated a noticeable discrepancy between the calculated dopant ratios presented in (Table 1) and the measured dopant ratios for the powders produced in Table 2. These findings indicate that not all the dopants added participated as a part of HA. The increase in the molar ratio of (Ca+Cu)/(P+Se+S), which is over 1.67, is due to the partial substitution of P ions by CO_3_ as confirmed by the FTIR results. This substitution is most likely the result of an ion exchange with atmospheric CO_2_ during the dissolving, stirring, and reaction phases.^33,36^ More research is necessary to determine the source of CO_2_ and the specific process of replacement because these issues fall outside the purview of the current work. However, the substitution of carbonate into the HA crystal structure causes a decrease in crystallinity and an increase in solubility.^37,38^ As well as the determined amounts of Se in the HA lattice were 2.58 mol% and 2.48 mol%, i.e., which was higher than the calculated value (1.97 mol%). While the amounts of Cu introduced into the HA lattice were 3.45 mol% and 3.35 mol%, i.e., less than the calculated value of (5 mol%) which means that Cu was loaded at amounts less than that of the feed values. A partial substitution of small amounts of Cu substituted the Ca ions in the HA crystal structure. This may be attributed to the smaller ionic radius of Cu^2+^ ions (0.073 nm) compared to that of Ca^2+^ ions (0.099 nm).^39^ It has been reported that SO_4_^2-^ ions substitute for PO_4_^3-^ ions within the apatite lattice, (sulphate ionic radius is larger than the phosphate one; 0.258 nm vs 0.238 nm).^22^ The incorporation of SO_4_^2-^ into apatite structure leads to increased structural disorder. SeO_4_^2-^ ions have a geometric shape that resembles that of PO_4_^3-^ ions, but they are much larger, measuring 0.249 nm as opposed to 0.238 nm for PO_4_^3-^ ions.^40–42^ SeO_3_2^-^ ions have a diameter of 0.239 nm, which is comparable to that of PO_4_^3-^ ions, but they are geometrically arranged differently and are composed of a flat trigonal pyramid structure.^41,43,44^ However, the recorded FTIR bands assigned to the SeO_3_^2-^ ions in the spectra of the apatite (SeHA), confirming their presence in the structure. The presence of the divalent selenium oxy-anion in the apatite structure, presumably as replacements for some of the PO_4_^3-^ ions in the apatite lattice, is to be expected given the charge compensation necessary for substitution of a -2 anion (SO_4_^2-^) for a -3 anion (PO_4_^3-^). The substitution of S ions is favoured in the presence of Se ions as evident from the recorded value (1.95 mol%) in SeCuHA, which is higher than that of CuHA (1.7 mol%). While slightly decreasing the incorporation amount of that have a small ionic radius, Cu ions (3.35 vs 3.45 mol%, in SeCuHA and CuHA, respectively).

**Table 2.** Elemental composition of different prepared powders measured by ICP-OES.

Samples	Se/(P+Se+S) (%)	Cu/(Ca+Cu) (%)	S/(P+Se+S) (%)	(Ca+Cu)/(P+Se+S) molar ratio	Ca/P molar ratio
HA	—	—	—	1.73	1.73
SeHA	2.58	—	—	1.74	1.79
CuHA	—	3.45	1.7	1.73	1.7
SeCuHA	2.48	3.35	1.95	1.72	1.74

Overall, the results suggest that the incorporation of dopants into the HA structure can be complex and influenced by several factors, including ionic radius, charge compensation, and structural disorder. Further studies may be necessary to understand the mechanisms underlying the incorporation of different dopants into the HA structure and their effects on the material's properties thoroughly.

**Figure 1.**
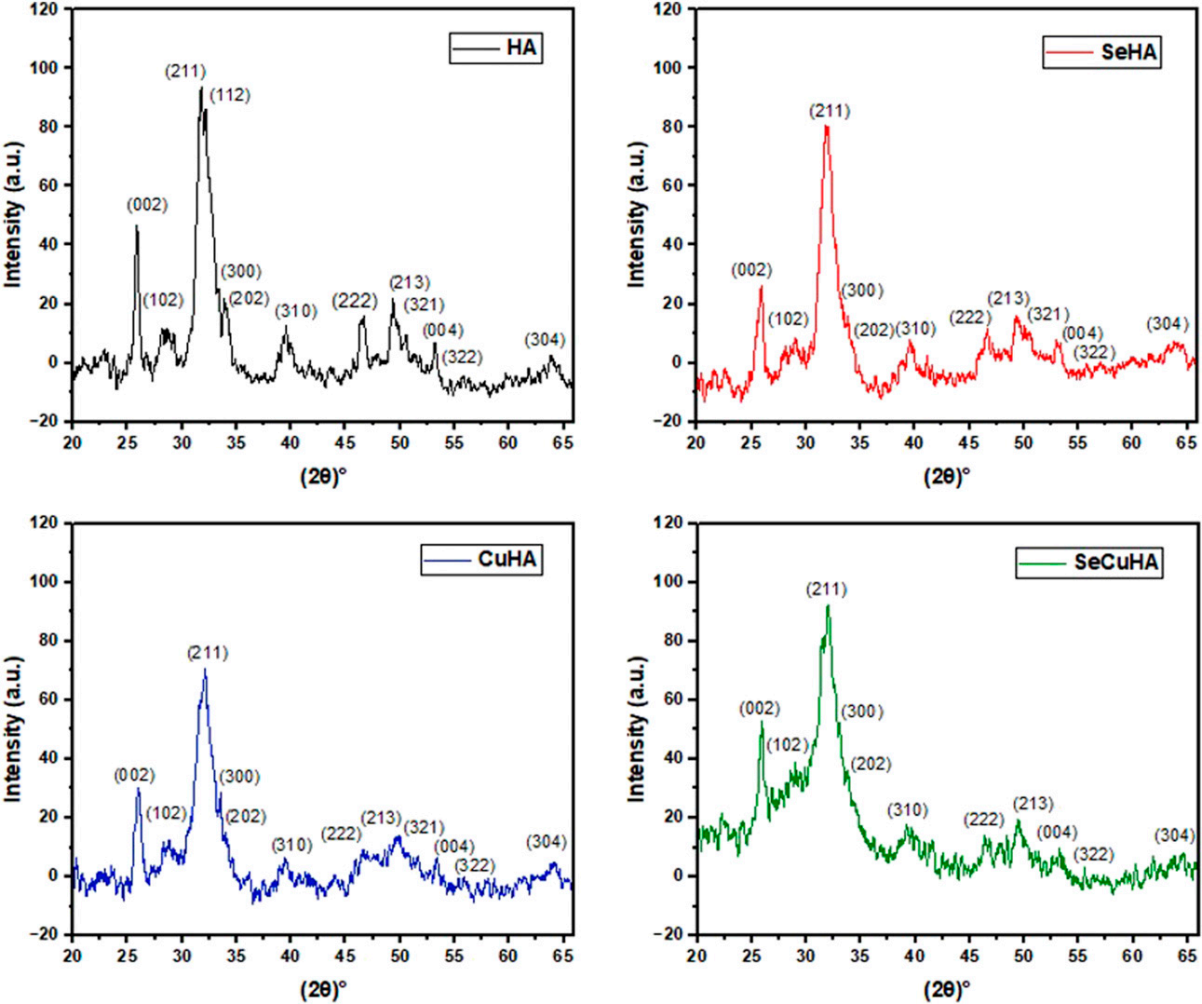
XRD spectra of prepared powders: HA, SeHA, CuHA and SeCuHA.

The XRD pattern of the prepared powders are shown in Figure 1. All peaks correspond well with the JCPDS PDF card no. 09-432 reference pattern for HA with no impurities. The pH of the reaction medium was maintained at 8.3, Ortiz et al. (2020) showed that the shape and size of the nanoparticles as well as the number of crystalline phases can change depending on the pH during the synthesis. When the pH value drops from 9.6 to 7, it has been seen that the production of the monoclinic phase increases and the hexagonal phase decreases, additionally, the crystallite size drops from 46.69 to 19.56 nm.^45^ Broadening of the peaks in the substituted hydroxyapatite, particularly in the CuHA and SeCuHA powders, compared to the pure phase indicated a decrease in the crystallite size of HA powders.^38,46,47^ The issue of generating a nano-sized HA has received a lot of attention since human bone HA is in the nanometer size range and because it functions better in clinical settings than micron-sized HA.^30^ These findings confirm the successful ion incorporation of copper and/or selenium into the HA crystal structure. The molar fractions of Cu^2+^ and Se^4+^ ions in the samples were relatively small compared to the original components of HA. Consequently, no new compounds were formed, which was evident from the absence of characteristic peaks.

**Figure 2.**
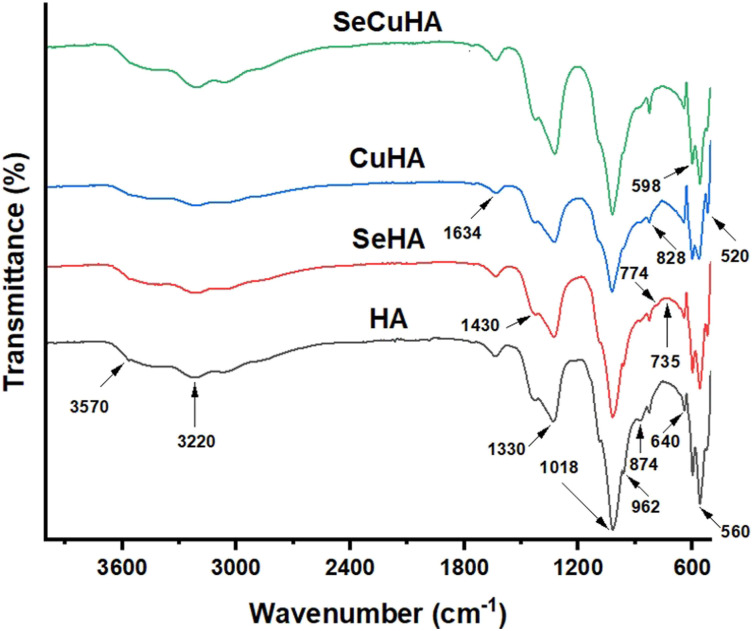
FTIR spectra of different hydroxyapatite powders: HA, SeHA, CuHA and SeCuHA.

FTIR spectra of the different apatite powders are displayed in Figure 2. There were typical absorption bands associated with phosphate bending modes of vibration at 520, 560, and 598 cm^−1^, symmetric stretching at 962 cm^−1^, and asymmetric stretching at about 1018 cm^−1^. There were also seen hydroxyl bands at 640 and 3570 cm^−1^. In the substituted HA, especially in the CuHA, the intensity and resolution of the bands observed for HA reduced. All the apatite powder spectra showed additional bands at 874, 1330, and 1430 cm^−1^ that were carbonates in the B position. Adsorbed water band at 3220 cm^−1^ and an occluded water band at 1634 cm^−1^ were seen in all the spectra. Two distinct shoulder and less intense bands at approximately 774 and 735 cm^−1^ respectively appeared in the spectra of SeHA samples. These bands were attributed to the vibrations of Se–O bands of the selenite ion (SeO_3_^2-^) in hydroxyapatite.^14,41,44^

**Figure 3.**
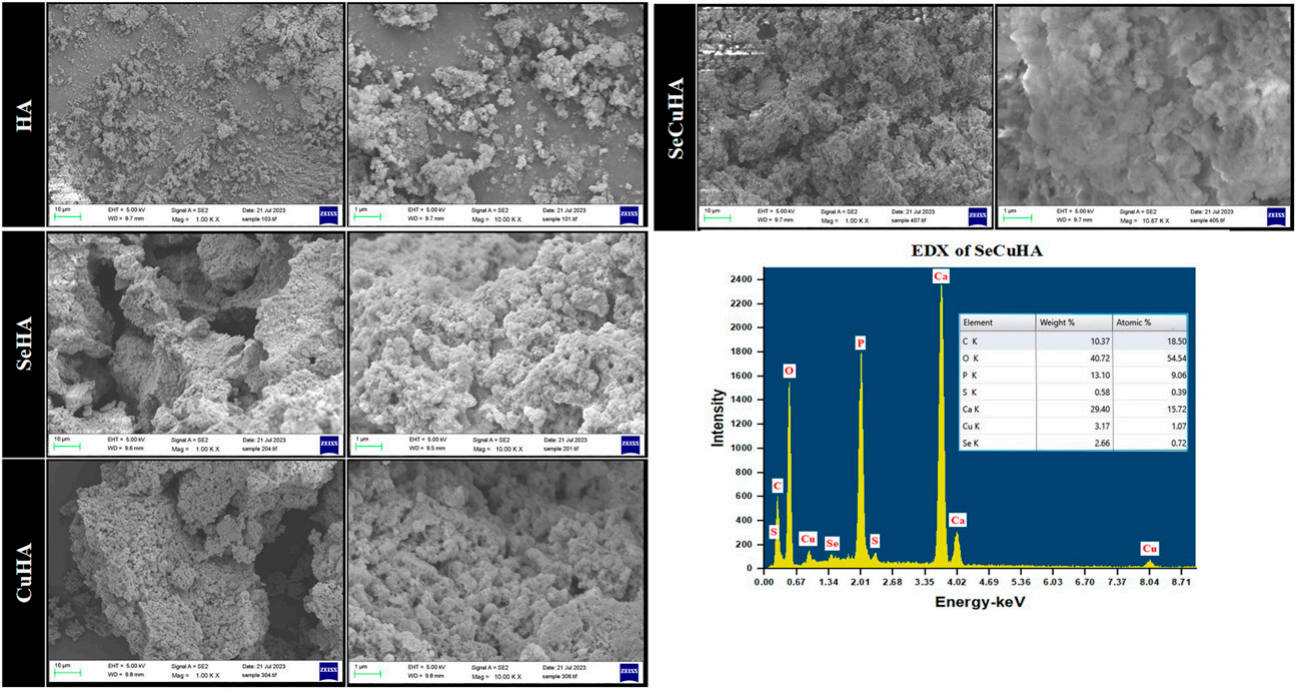
FESEM images of prepared powders: hydroxyapatite (HA), selenium substituted hydroxyapatite (SeHA), copper substituted hydroxyapatite (CuHA) and selenium and copper substituted hydroxyapatite (SeCuHA), at two magnifications of 1 and 1000 KX. EDX analysis for SeCuHA powders showed its elemental composition.

The FESEM images obtained at the two magnifications (1.00 and 10.00 KX) revealed that the hydroxyapatite powders are made up of clusters of tiny particles that appear to be spherical in shape (Figure 3). This agglomeration might be connected to the preparation’s 24-h stirring period.^48^ Furthermore, a considerable tendency to produce thick agglomerates is visible in the substituted hydroxyapatite creating pores within, as seen at 10.00 KX magnification. Furthermore, when ions were doped, more microscale aggregates were seen, most likely as a result of ion exchange in the material, where coarsening of crystallite size happens in the particles to form denser structures as microscale aggregates. Particle fusion also leads to the formation of loose aggregates.^49,50^ However, because of the dense formed clusters of both pure and doped HA particles, determining the size of a single particle was challenging. Spherical powders offer higher rheological qualities than irregular powders and less cytotoxicity than needle and plate shaped nanohydroxyapatite, making them ideal for medical applications.^51,52^ Elements Ca, P, Cu, Se, S, and O were discovered in these SeCuHA agglomerates using the EDX method, which agrees with the ICP-OES results (Table 2).

Based on the ICP-OES, XRD, FTIR and SEM-EDX results, it is observed that Se and/or Cu containing HA showed pure phases of HA without any impurities in addition to the doping of S besides Cu ions in the HA lattice indicated the dual substitution caused by CuSO_4_.5H_2_O. All these elements have a vital role in the cell growth and proliferation of the cells as well as the antibacterial activity effect.^10–14,18,21,53,54^ The occurrence of S^2-^ ion in the hydroxyapatite was detected by ICP-OES in copper containing HA powders samples accompanying substitution of Cu^2+^ in the lattice indicated the dual substitution might have been caused by CuSO_4_.5H_2_O, the precursor used.

The chemical composition and surface topography of a nanostructured material have a significant impact on cytocompatibility performance of the material. These factors also control how well cells adhere to surfaces, migrate, differentiate, and proliferate. Thus, the particle diameter, shape, size, dose, and contact properties of nanohydroxyapatite have an impact on its biotoxicity.^55^ The crystal structure of natural hydroxyapatite can be altered by various ionic substitutions.

**Figure 4.**
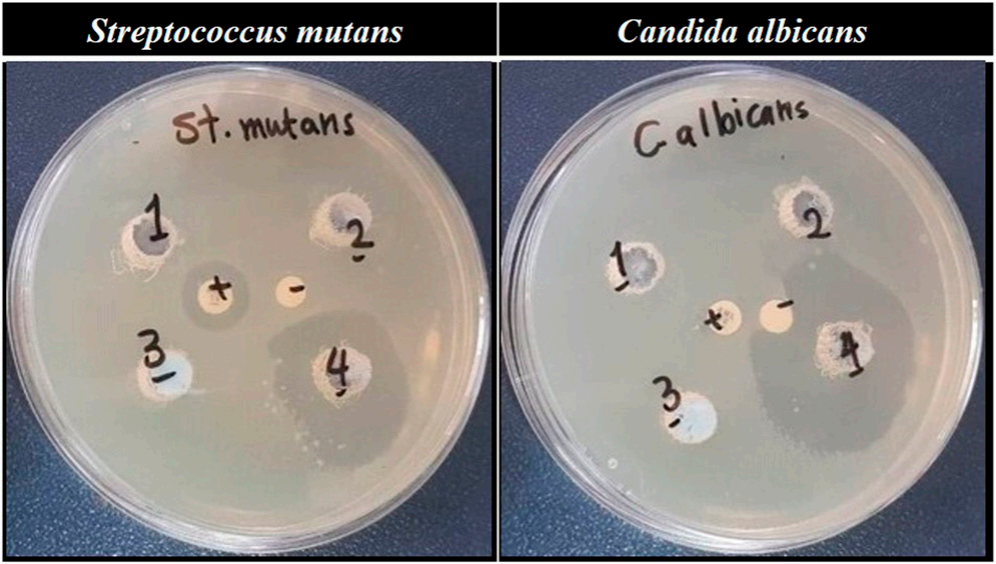
Photographs of the antimicrobial activity of apatite samples against the tested microbes. (1) HA, (2) SeHA, (3) CuHA, (4) SeCuHA. Ciprofloxacin and distilled water as positive and negative controls respectively are located in the centre.

The well diffusion technique was used to test the ability of the four prepared apatite samples to inhibit microbial growth. The diameter of the zone of inhibition formed around the wells was measured. SeCuHA sample showed excellent antimicrobial properties, in contrast to the rest of samples, against both tested microorganisms even though the zone of inhibition was extensively dispersed throughout the wells, as shown in Figure 4. It is measured as 25 ± 0.6 mm and 32 ± 0.41 mm against *Streptococcus mutans* and *Candida albicans*, respectively in contrast to the positive control which showed zone of diameter equals 19 ± 0.28 mm against *Streptococcus mutans*. That is may due to the release of the Se^4+^, Cu^2+^ and S^2-^ ions inhibit the growth of the surrounding microbes and enhance the antimicrobial activity of the apatite sample.

**Figure 5.**
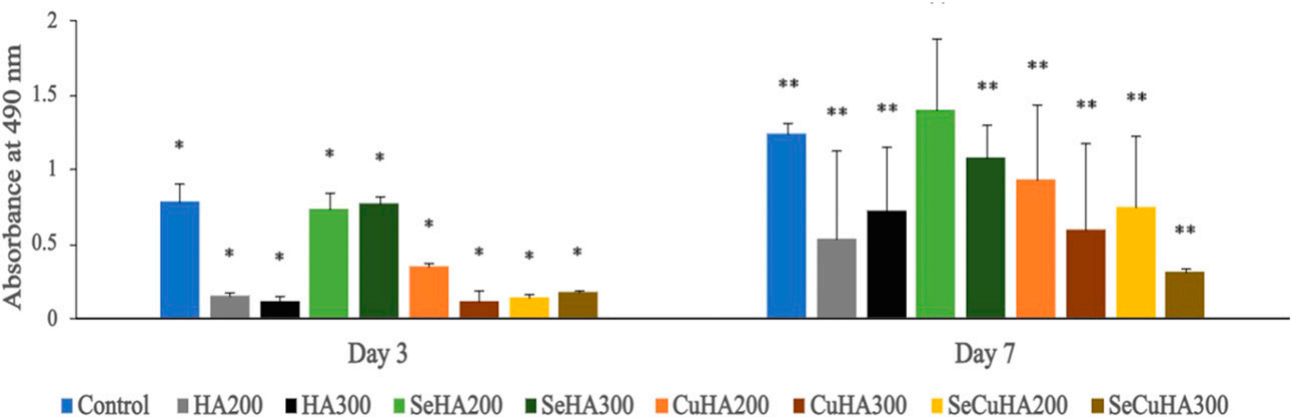
Cell metabolic activity of MC3T3-E1 cells cultured on tissue culture plate (control) and different HA powders samples after incubation for 3 and 7 days. Data are represented as mean SD (*n* = 3) with statistical assessment performed by using the one-way ANOVA with Bonferroni’s Multiple Comparison Post Test and Holm Comparison Test. All pairs simultaneously compared, and they are significantly differenced with the *p* value <0.01 over each the day 3 (*) and day 7 (**).

The effect of the hydroxyapatite powders (HA, SeHA, CuHA and SeCuHA) at two different concentrations (200 and 300 μg/mL) on MC3T3-E1 were tested. Figure 5 displayed the cell compatibility by measuring the mitochondrial activity of the MC3T3-E1 cells using the MTS assay. A significant difference (*p*<0.01) was detected in between all powder samples and the control (cells only on TCP). By comparing the data of MTS it can be determined that the MC3T3-E1 cells after cultured for 7 days with selenium substituted hydroxyapatite (SeHA) at both concentrations (200 and 300 μg/mL), copper substituted hydroxyapatite (CuHA) at concentration (200 μg/mL) and selenium and copper substituted hydroxyapatite (SeCuHA) at concentration (200 μg/mL) showed the highest metabolic activity rate than the other powders in the descending order: SeHA200, SeHA300, CuHA200 then SeCuHA200.

**Figure 6.**
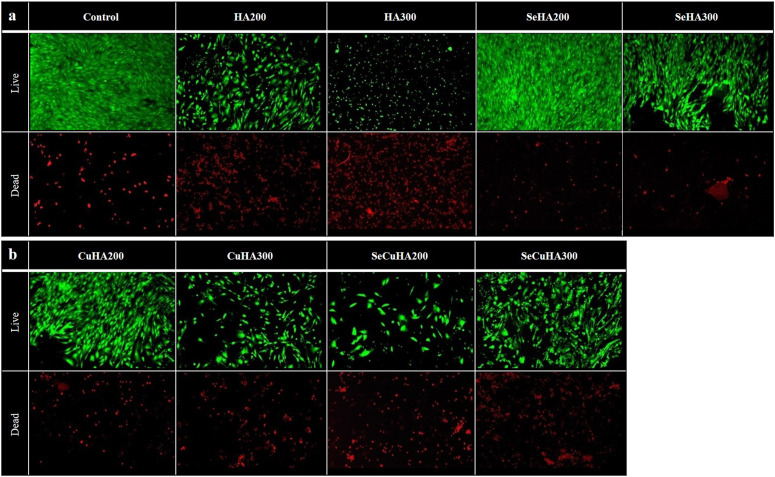
(a) and (b) fluorescence microscopy images of control and different HA powders with MC3T3-E1 cells cultured for 7 days (after live/dead assay). Living cells are stained green, dead cells are stained red.

The fluorescence images following the live-dead assay for the MC3T3-E1 cells cultured on various hydroxyapatite powders for 7 days (Figure 6) revealed that SeHA at both concentrations (200 and 300 μg/mL) and CuHA at low concentration (200 μg/mL) showed a higher accumulation of live cells and lower accumulations of dead cells than all other powders. Even at low concentration of SeHA (200 μg/mL), more live cells were present than in the control, demonstrating that selenium at this concentration promotes cell proliferation.

Moreover, the results showed that CuHA at a high concentration of 300 μg/mL and SeCuHA at a low concentration of 200 μg/mL performed better than the unsubstituted HA by comparing abundance of live cells to the dead cells. The incorporation of Se and Cu, in addition to S ions into the hydroxyapatite lattice is likely responsible for this improvement. These ions are essential for cell development and proliferation,^53,54,56^ and their incorporation may have a positive impact on the cytocompatibility of the hydroxyapatite powders.

The capacity of these materials to induce cell adhesion and spreading, their cytocompatibility and antimicrobial activity make it a promising candidate for further investigation in the field of biomedical materials. However, further research is needed to determine the long-term effects of SeCuHA on cell behaviour and tissue regeneration.

## Conclusion

In conclusion, the substitution of different ions, including selenium, copper, and sulphur, into the hydroxyapatite lattice affects the properties of the resulting material. In this study, hydroxyapatite powders substituted with these ions were synthesized by an aqueous precipitation method and using the freeze-drying technique. The XRD peaks became broader and all XRD and FTIR patterns referred to HA with no impurities, indicating the incorporation of the substituting ions. Selenium-substituted hydroxyapatite (SeHA) was found to increase the proliferation of MC3T3-E1 cells, while SeCuHA, which contained three substituted ions of Se, Cu and S, showed excellent antimicrobial activity against *Streptococcus mutans* and *Candida albicans*, as well as presenting cytocompatibility in vitro*.* These results suggest that SeCuHA has the potential to be used in various biomedical applications, including orthopaedic and dental applications, tissue engineering, and as a restorative dental material in toothpaste or as a filler or insert. Future studies could focus to evaluate the performance of SeCuHA in vivo to further establish its suitability for these applications.
